# COVID-19 vaccination efficacy in numbers including SARS-CoV-2 variants and age comparison: a meta-analysis of randomized clinical trials

**DOI:** 10.1186/s12941-022-00525-3

**Published:** 2022-07-03

**Authors:** Marharyta Sobczak, Rafał Pawliczak

**Affiliations:** Department of Immunopathology, Faculty of Medicine, Division of Biomedical Science, Medical University of Lodz, st. Zeligowskiego 7/9, 90-752, Lodz, Poland

**Keywords:** COVID-19, Vaccine, SARS-CoV-2, Coronavirus infection, Meta-analysis

## Abstract

**Background:**

New vaccines are being developed to fight the ongoing COVID-19 pandemic. In our study we compared the efficacy of COVID-19 vaccines to prevent COVID-19-related infections and mortality.

**Methods:**

17 randomized clinical trials of COVID-19 vaccines were included after search in databases. We compared COVID-19 vaccines based on symptomatic and severe infections, number of deaths and hospitalizations related to COVID-19. Also, we analyzed the efficacy of COVID-19 against different variants of SARS-CoV-2 as well as according to different age groups. Random effects model using Mantel–Haenzeal method was used to pool relative risk (RR).

**Results:**

Our meta-analysis shows that full vaccination could decrease not only the risk of symptomatic or severe COVID-19, the risk of hospitalization and death caused by COVID-19. COVID-19 vaccines were also effective against variants of SARS-CoV-2 (RR = 0.36; 95% CI [0.25; 0.53], p < 0.0001). However, efficacy of vaccination varied in COVID-19 variant-dependent manner. Moreover, the analysis in different age groups showed that COVID-19 vaccines had the similar results: the risk was slightly lower in adults compared to elderly cohort $(\ge$ 65 years): (RR = 0.16, 95% CI [0.11; 0.23]) and (RR = 0.19, 95% CI [0.12; 0.30]), respectively.

**Conclusions:**

Data obtained from clinical trials of COVID-19 vaccines looks promising, in order to fully investigate efficacy of the vaccines further clinical examination is required especially considering new SARS-CoV-2 variants.

**Supplementary Information:**

The online version contains supplementary material available at 10.1186/s12941-022-00525-3.

## Introduction

Since the start of the COVID-19 (coronavirus disease 2019) pandemic, when the first case was identified in Wuhan in December 2019, the whole world has been focused on developing an effective vaccine to fight the pandemic. The global pandemic was caused by novel coronavirus called SARS-CoV-2. This enveloped virus has single-stranded positive-sense RNA genome and belongs to family *Coronaviridae* [1, 2]. Coronaviruses can infect animals and humans, causing mild or severe acute respiratory infections. Two coronaviruses (SARS-CoV—severe acute respiratory syndrome coronavirus, and MERS-CoV—Middle East respiratory syndrome coronavirus) have already caused epidemics in 2002 and 2012. Interestingly, genome sequence of a new SARS-CoV-2 is similar in 50% with MERS-CoV and in 79% with SARS-CoV genomes [3].

COVID-19 may cause different symptoms such as dry cough, loss of smell and dyspnea, fever and fatigue, with incubation period around 5.2 days. In severe COVID-19 cases, symptoms may escalate to pneumonia and even severe acute respiratory distress syndrome and death [4, 5]. However, the novel variants of SARS-CoV-2 have shorter incubation period, for example for B.1.617.2 variant incubation period equals 4 days [6]. According to WHO (World Health Organization) COVID-19 dashboard [7] on the day of December 3, 2021 from 263.56 mln confirmed COVID-19 cases, more than 5.23 mln deaths were recorded. Moreover, around 7.86 billion doses of vaccines have been applied.

In order to effectively control COVID-19 pandemic, vaccination, that can stimulate both adaptive and innate immune responses, may be applied. Nowadays, there are many vaccines against COVID-19, which are tested in clinical trials. These vaccines can be divided into few types: DNA or mRNA vaccines, viral-vector based vaccines, subunit vaccines and inactivated or attenuated vaccines [8, 9]. WHO recommendations towards the COVID-19 vaccines highlight, that minimum criterion for the vaccine candidate to be acceptable is to reach ~ 50% point estimate efficacy in prevention of disease including its severe form, as well as spread of the virus. Of note, that vaccine candidate might prove useful in fight against COVID-19 even if not all of those endpoints are met. In turn, FDA (Food Drug Administration) suggests that a key feature of those candidates is to reach the 50% endpoint estimate in clinical study including placebo group. Unfortunately, even large population tested in phase 3 clinical trials, might be not enough to assess the vaccine’s efficiency and further either phase 4 trials or epidemiological studies are required in order to increase the size of tested population [10]. In this study, we would like to conduct systematic review and meta-analysis of data gathered by RCTs (randomized controlled trials) assessing COVID-19 vaccine efficacy. Analyzed COVID-19 vaccines had been evaluated basing on their efficacy measured by number of variables referring to the numbers of: symptomatic COVID-19 cases, severe COVID-19, hospitalization and death cases compared to the control group. Additionally, we analyzed COVID-19 vaccines against different variants of SARS-CoV-2 as well as in different age groups. Analysis show, that vaccines efficiently protect from severe symptoms development and COVID-19-related death and hospitalization in vaccinated patients.

## Methods

### Search strategy

The systematic review and meta-analysis were conducted according to the Preferred Reporting Items for Systematic Reviews and Meta-Analyses (PRISMA) guidelines [11]. Embase, PubMed and the Cochrane Central Register of Controlled Trials databases were searched to find literature published before November 2, 2021. The following search strategy was used: *(((((COVID-19) OR (coronavirus infection)) OR (SARS-CoV-2)) OR (coronavirus)) AND ((vaccine) OR (vaccination))) AND (efficacy).*

### Study selection and data extraction

Inclusion criteria referred to articles of blinded control-compared RCTs of COVID-19 vaccines; while excluding criteria: articles not written in English, as well as not containing endpoints, such as: number of symptomatic COVID-19 cases, number of severe cases of COVID-19, number of hospitalizations and deaths related to COVID-19, as well as number of COVID-19 cases belonging to different SARS-CoV-2 lineages in experimental and control groups after full vaccination.

### Quality assessment

The quality of trials was evaluated according to the Cochrane Collaboration’s tool for assessing risk of bias in randomized trials [12], using the following criteria: random sequence generation, allocation concealment, blinding of participants and personnel, blinding of outcome assessment, incomplete outcome data, selective reporting and other bias. For each criteria, risk of bias was assessed at 3 levels: low, high or unclear risk.

### Statistical analysis

Statistical analysis of data was prepared in R (version 4.0.3). To compare the efficacy of COVID-19 vaccination in experimental group compare to control, the relative risk (RR) with 95% confidence interval (CI) was used for dichotomous outcomes. Random effects model using Mantel–Haenzeal method was used to calculate effect sizes. I^2^ statistics was used to evaluate the heterogeneity of studies: I^2^ < 40% may not be important; 30% < I^2^ < 60% means moderate heterogeneity; 50% < I^2^ < 90% means substantial heterogeneity; I^2^ > 75% means considerable heterogeneity [13]. To assess publication bias, funnel plot and Peters’ regression test were used. Results of this meta-analysis were considered statistically significant at p < 0.05.

## Results

### Search results

**Fig. 1:**
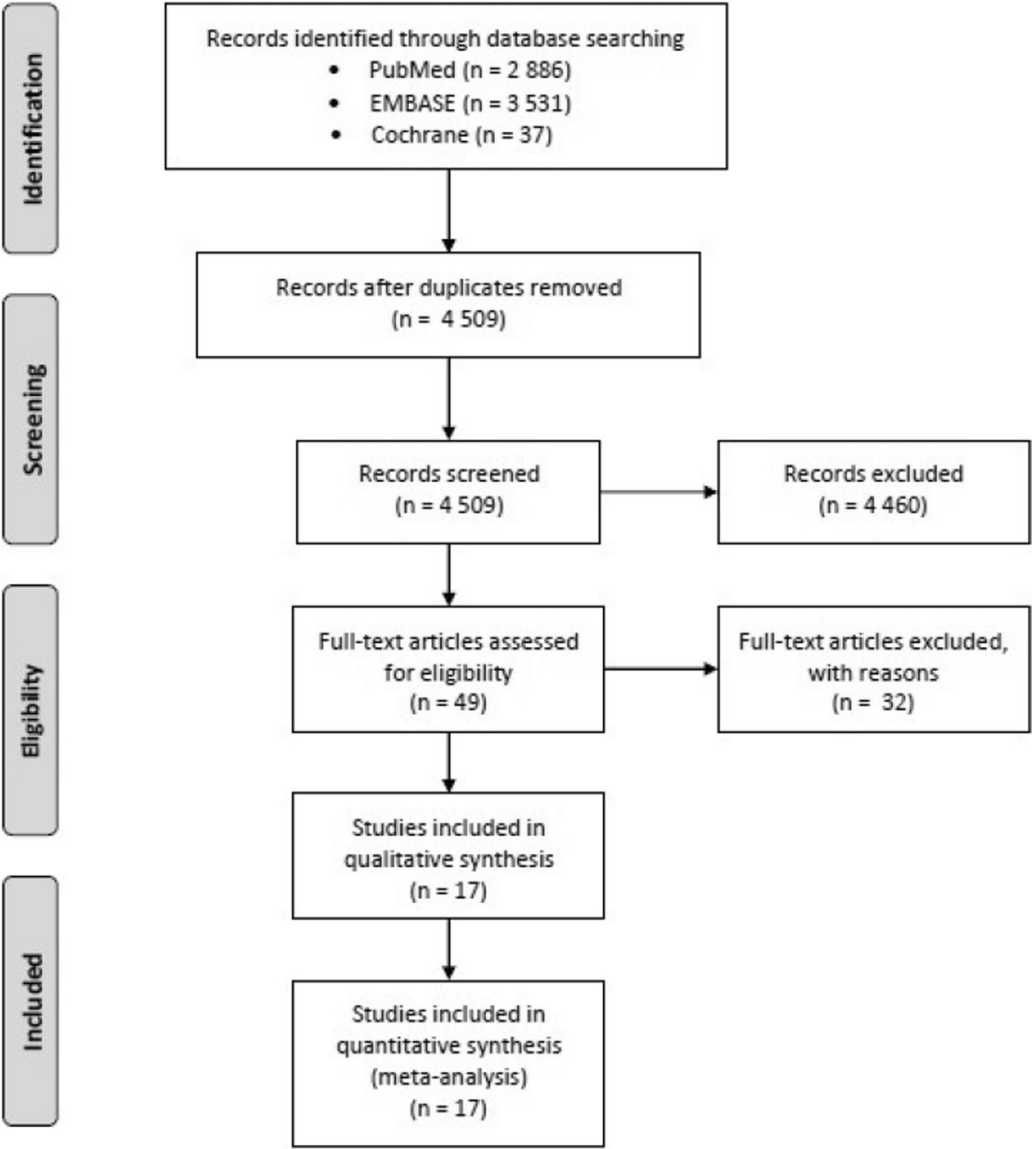
Study selection for meta-analysis

Literature search detected 4509 articles after removal of duplicates (Fig. 1). During screening of titles and abstracts, we excluded 4460 articles, such as reviews and meta-analysis, in vitro studies, studies on animals and humans, such as case reports and observational studies. Moreover, we excluded articles not written in English, as well as comments, recommendations and expert opinions. After full-text assessment, 17 articles were included for quality and quantity analysis.

All included studies are randomized controlled trials with control group. In the studies, four types of vaccines were evaluated: mRNA vaccines [14–19], viral vector vaccines [20–25], subunit [26, 27] and inactivated vaccines [28–30]. Among these trials, in Brazilian study by Clemens et al. [23] and by Voysey et al. [25] at first dose participants received MenACWY conjugate vaccine as a control, while at second dose they received placebo as a control, whereas in the study from the United Kingdom by Voysey et al. [25] there were two experimental cohorts: in first cohort the participants received low dose of vaccine at first, and a standard dose as a second dose. While in the second cohort, participants received two standard doses, and both control groups received MenACWY conjugate vaccine. Two studies were conducted in South Africa [22, 26], one study in South Africa, Brazil, and the United Kingdom [25], one study in Indonesia [29], one study in Russia [20], one study in Brazil [23], one in the United Kingdom [27], one in Turkey [30], one in the United States, Chile, and Peru [24], one in South Africa, Argentina, Chile, Brazil, Colombia, Peru, Mexico, and the United States [21], one in the United Arab Emirates, Jordan, Egypt and Bahrain [28], one in the United States, Argentina, Brazil, South Africa, Germany, and Turkey [18], and five in the United States [14–17, 19]. Additionally, three studies were conducted on adolescents [17–19]. Table 1 shows the characteristics of included studies.

**Table 1:** Characteristics of included RCTs

Studies	Blinding	Vaccines	Number of participants that received the first dose	Number of participants that received the second dose (seronegative, used to efficiency calculation)	Mean age [years]	Age	Sex [% of male]	Phase	Time between doses [days]	Observation time (used to efficiency calculation)
*mRNA vaccines*										
Baden et al. 2021 [12]	Observer-blinded	mRNA-1273	VG: (n = 15,181) PG: (n = 15,170)	VG: (n = 14,134) PG: (n = 14,073)	51.4	$\ge$ 18 years	52.7%	3	28	At least 14 days after the second dose
El Sahly et al. 2021 [13]	Part A: observer-blinded Part B: open-label	mRNA-1273	VG: (n = 15,180) PG: (n = 15,166)	VG: (n = 14,287) PG: (n = 14,264)	51.4	$\ge$ 18 years	52.6%	3	28	At least 14 days after the second dose
Ali et al. 2021 [14]	Observer-blinded	mRNA-1273	VG: (n = 2486) PG: (n = 1240)	VG: (n = 2139) PG: (n = 1042)	14.3	12–17 years	51%	2/3	28	At least 14 days after the second dose
Polack et al. 2020 [11]	Observer-blinded	BNT162b2	VG: (n = 18,860) PG: (n = 18,846)	VG: (n = 18,198) PG: (n = 18,325)	52	$\ge$ 16 years	50.6%	2/3	21	At least 7 days after the second dose
Thomas et al. 2021 [15]	Observer-blinded	BNT162b2	VG: (n = 22,030) PG: (n = 22,030)	VG: (n = 20,998) PG: (n = 21,096)	51	$\ge$ 16 years//12–15 years	51%	2/3	21	At least 7 days after the second dose
Frenck et al. 2021 [16]	Observer-blinded	BNT162b2	VG: (n = 1131) PG: (n = 1129)	VG: (n = 1005) PG: (n = 978)	13.6	12–15 years	51%	3	21	At least 7 days after the second dose
*Viral vector vaccines*										
Voysey et al. 2021 [22]	Single-blind	ChAdOx1 nCoV-19	NA	VG: (n = 1367) CG: (n = 1374)	*NA*	18–55 years	VG: 35.2% CG: 32.5%	2/3	4–6 weeks	At least 14 days after the second dose
	Single-blind	ChAdOx1 nCoV-19	NA	VG: (n = 2377) CG: (n = 2430)	*NA*	$\ge$ 18 years	VG: 42% CG: 40%	2/3	4–6 weeks	At least 14 days after the second dose
	Single-blind	ChAdOx1 nCoV-19	NA	VG: (n = 2063) CG: (n = 2025)	*NA*	$\ge$ 18 years	VG: 38.9% CG: 42.9%	3	up to 12 weeks	At least 14 days after the second dose
Madhi et al. 2021 [19]	Double-blind	ChAdOx1 nCoV-19	VG: (n = 1011) PG: (n = 1010)	VG: (n = 750) PG: (n = 717)	30	18–65 years	56.5%	1b/2	21 to 35	At least 14 days after the second dose
Clemens et al. 2021 [20]	Single-blind	ChAdOx1 nCoV-19	*NA*	VG: (n = 4772) CG: (n = 4661)	*NA*	$\ge$ 18 years	VG: 44% CG: 46%	3	between 4 and 12 weeks	At least 15 days after the second dose
Falsey et al. 2021 [21]	Double-blind	ChAdOx1 nCoV-19	VG: (n = 21,583) PG: (n = 10,796)	VG: (n = 17,662) PG: (n = 8550)	50.2	18–64 years and $\ge$ 65 years	55.6%	3	4 weeks apart	At least 15 days after the second dose
Sadoff et al. 2021 [18]	Double-blind	Ad26.COV2.S	VG: (n = 21,895) PG: (n = 21,888)	–	52	$\ge$ 18 years	54.9%	3	–	At least 14 days after administration
										At least 28 days after administration
Logunov et al. 2021 [17]	Double-blind	rAd26 and rAd5	VG: (n = 16,427) PG: (n = 5435)	VG: (n = 14,964) PG: (n = 4902) *no information about serostatus*	45.3	$\ge$ 18 years	VG: 61.1% PG: 61.5%	3	21	At least 21 days after the first dose (day of dose 2)
*Inactivated vaccines*										
Al Kaabi et al. 2021 [25]	Double-blind	WIV04 and HB02	WIV04 group (N = 13,459), HB02 group (N = 13,465), in the alum-only group control (N = 13,458)	WIV04 group (N = 12,743), HB02 group (N = 12,726) and aluminum hydroxide (alum)–only control (N = 12,737)	36.1	$\ge$ 18 years	84.4%	3	21	At least 14 days after the second dose
Fadlyana et al. 2021 [26]	Double-blind	CoronaVac	VG: (n = 811) PG: (n = 809)	VG: (n = 798) PG: (n = 804)	35.5	18–59 years	64.5%	3	14	At least 14 days after the second dose
Tanriover et al. 2021 [27]	Double-blind	CoronaVac	VG: (n = 6646) PG: (n = 3568)	VG: (n = 6559) PG: (n = 3470)	45	18–59 years	57.8%	3	14	At least 14 days after the second dose
*Subunit vaccines*										
Shinde et al. 2021 [23]	Observer-blinded	NVX-CoV2373	VG: (n = 2199) PG: (n = 2188)	HIV-negative participants: VG: (n = 1281) PG: (n = 1255)	32	18–84 years	57%	2a/b	21	At least 7 days after the second dose
Heath et al. 2021 [24]	Observer-blinded	NVX-CoV2373	VG: (n = 7569) PG: (n = 7570)	VG: (n = 7020) PG: (n = 7019)	56	18–84 years	51.6%	3	21	At least 7 days after the second dose

*VG* vaccine group, *PG* placebo group, *CG* control group, *NA* not available

### Quality assessment

Risk of bias was prepared for 17 included RCTs. According to our risk of bias assessment, 2 of the analyzed studies represent high risk of bias; while remaining 15 studies represent low risk of bias. Additional file 1 shows the summary of risk of bias.

### The efficacy of vaccines against symptomatic COVID-19 infections

**Fig. 2:**
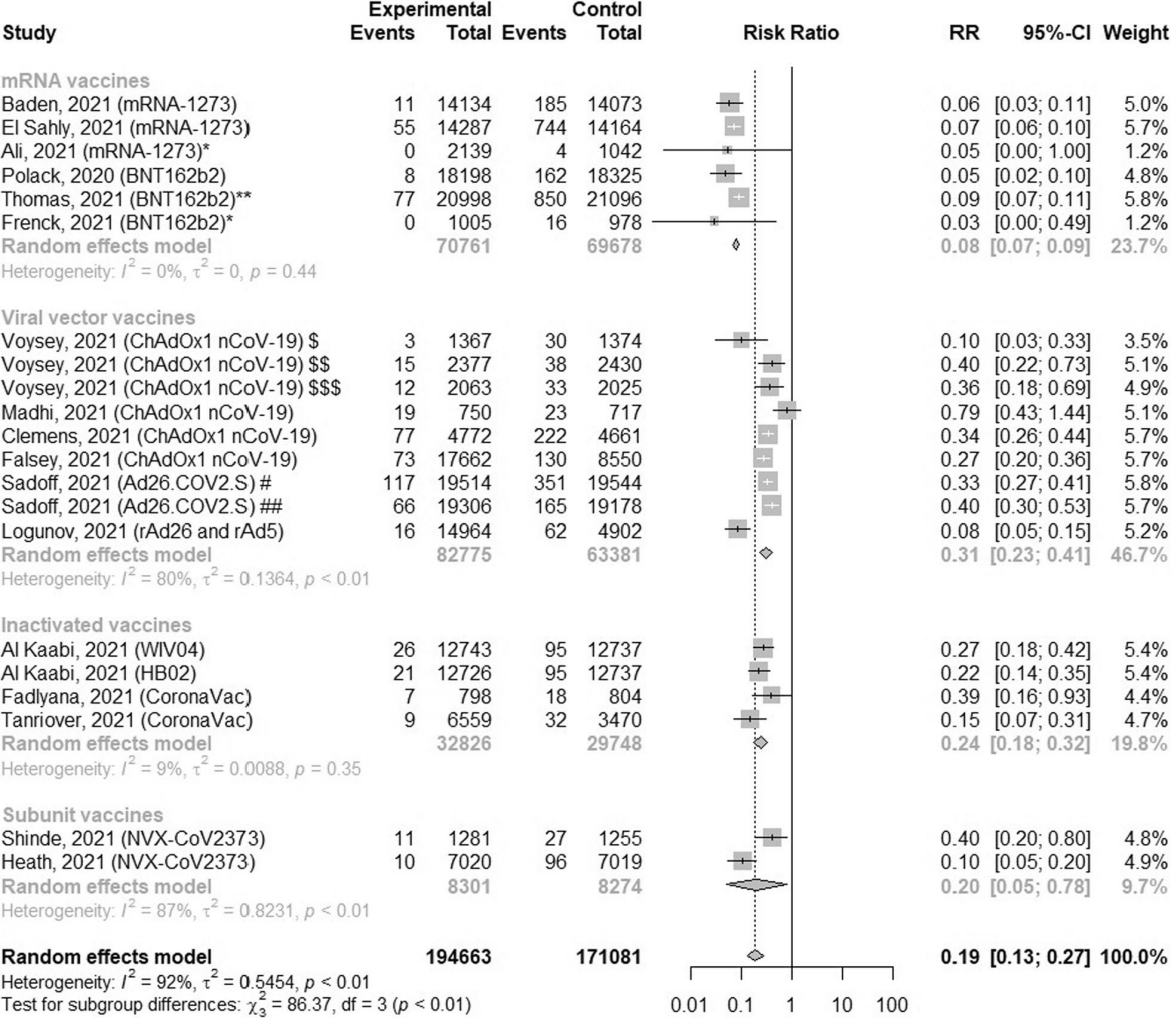
The efficacy of COVID-19 vaccines for preventing symptomatic COVID-19. *Studies in adolescents; **study in adolescents and adults; ^$^first dose was low, while second dose was standard, the United Kingdom; ^$$^both doses were standard, the United Kingdom; ^$$$^both doses were standard, Brazil; ^#^observation at least 14 days after vaccination; ^##^observation at least 28 days after vaccination

Because of high level of heterogeneity, the subgroup analysis of symptomatic COVID-19 incidences from clinical trials of different types of vaccines compared to control was performed (Fig. 2). The analysis found that vaccination decreased the risk of symptomatic COVID-19 infection by 81% (RR = 0.19; 95% CI [0.13; 0.27]; p < 0.0001). The lowest level of risk of symptomatic COVID-19 infection was noted after full vaccination with mRNA vaccines and equals 0.08 (95% CI [0.07; 0.09]) without heterogeneity, while in case of viral vector vaccines the risk was 0.31 with considerable heterogeneity (95% CI [0.23; 0.41], I^2^ = 80%). Similar effects were obtained after vaccination with inactivated and subunit vaccines: 0.24 (95% CI [0.18; 0.32], I^2^ = 9%) and 0.20 (95% CI [0.05; 0.78], I^2^ = 87%), respectively. However, the risk of symptomatic COVID-19 after vaccination with one dose vaccine (Ad26.COV2.S) was higher after 28 days after vaccination than after 14 days following the vaccination: 0.33 (95% CI [0.27; 0.41]) and 0.40 (95% CI [0.30; 0.53]), respectively. In adolescents, full vaccination with mRNA-1273 vaccine decreased the risk of symptomatic COVID-19 infection by 95% (RR = 0.05, 95% CI [0.00, 1.00]), while BNT162b2 vaccine decreased by 97% (RR = 0.03, 95% CI [0.00, 0.49]).

### The efficacy of vaccines against severe COVID-19 infections

**Fig. 3:**
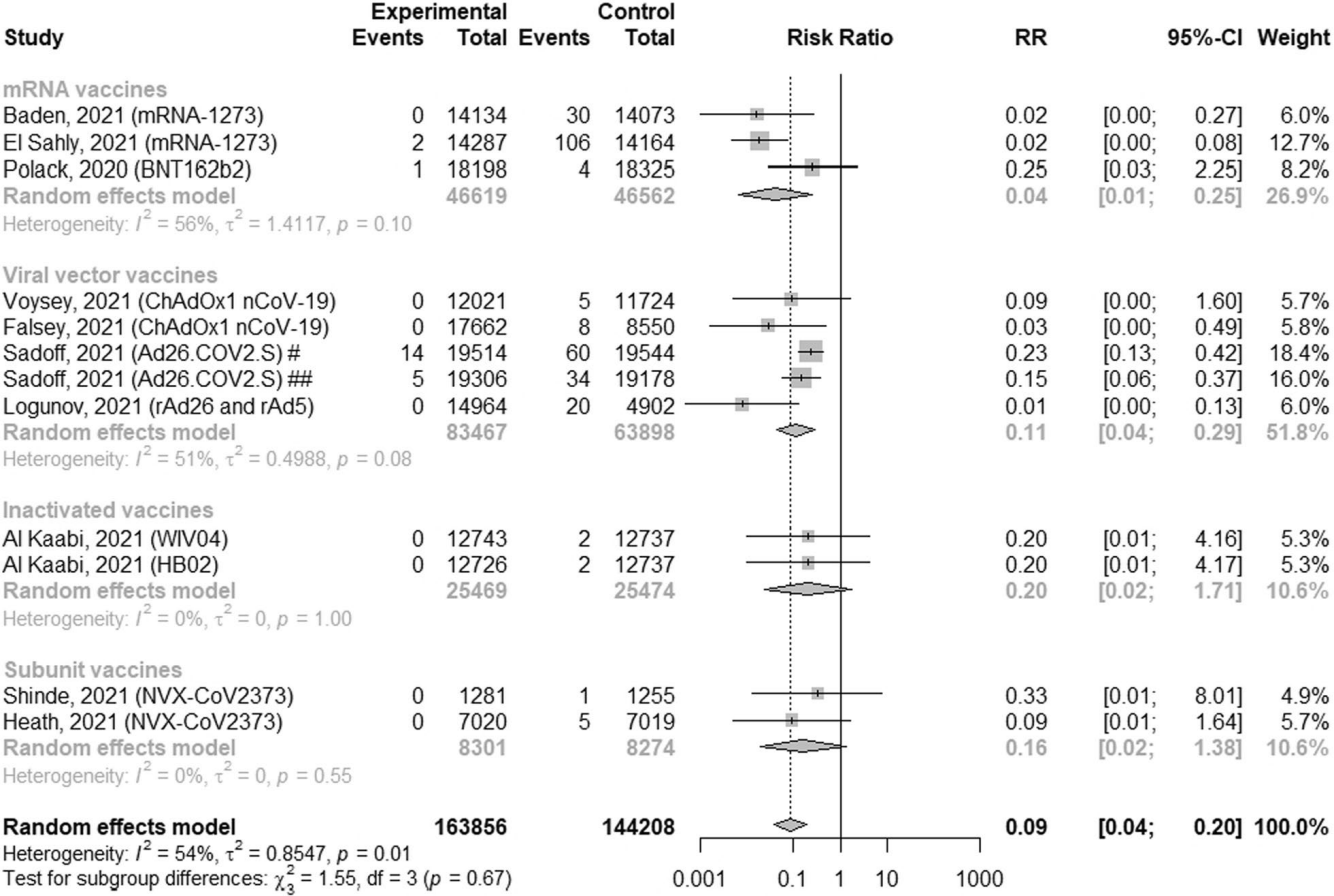
The efficacy of COVID-19 vaccines for preventing severe COVID-19. ^#^Observation at least 14 days after vaccination; ^##^observation at least 28 days after vaccination

Only 10 studies were included in the subgroup analysis, because other studies reported no cases of severe COVID-19. (Fig. 3). Overall, full COVID-19 vaccination decreased the risk of severe COVID-19 infection by 91% (RR = 0.09; 95% CI [0.04; 0.20]; p < 0.0001, I^2^ = 54%). Among vaccines, the lowest risk of severe infection course of COVID-19 was 0.04 (95%CI [0.01; 0.25]) after vaccination with mRNA vaccines. Moreover, the risk of severe COVID-19 infection with one dose vaccine (Ad26.COV2.S) was lower after 28 days after vaccination compared to risk after 14 days following the vaccination: 0.15 (95% CI [0.06; 0.37]) and 0.23 (95% CI [0.13; 0.42]), respectively.

### The efficacy of vaccines against hospitalization related with COVID-19 infections

**Fig. 4:**
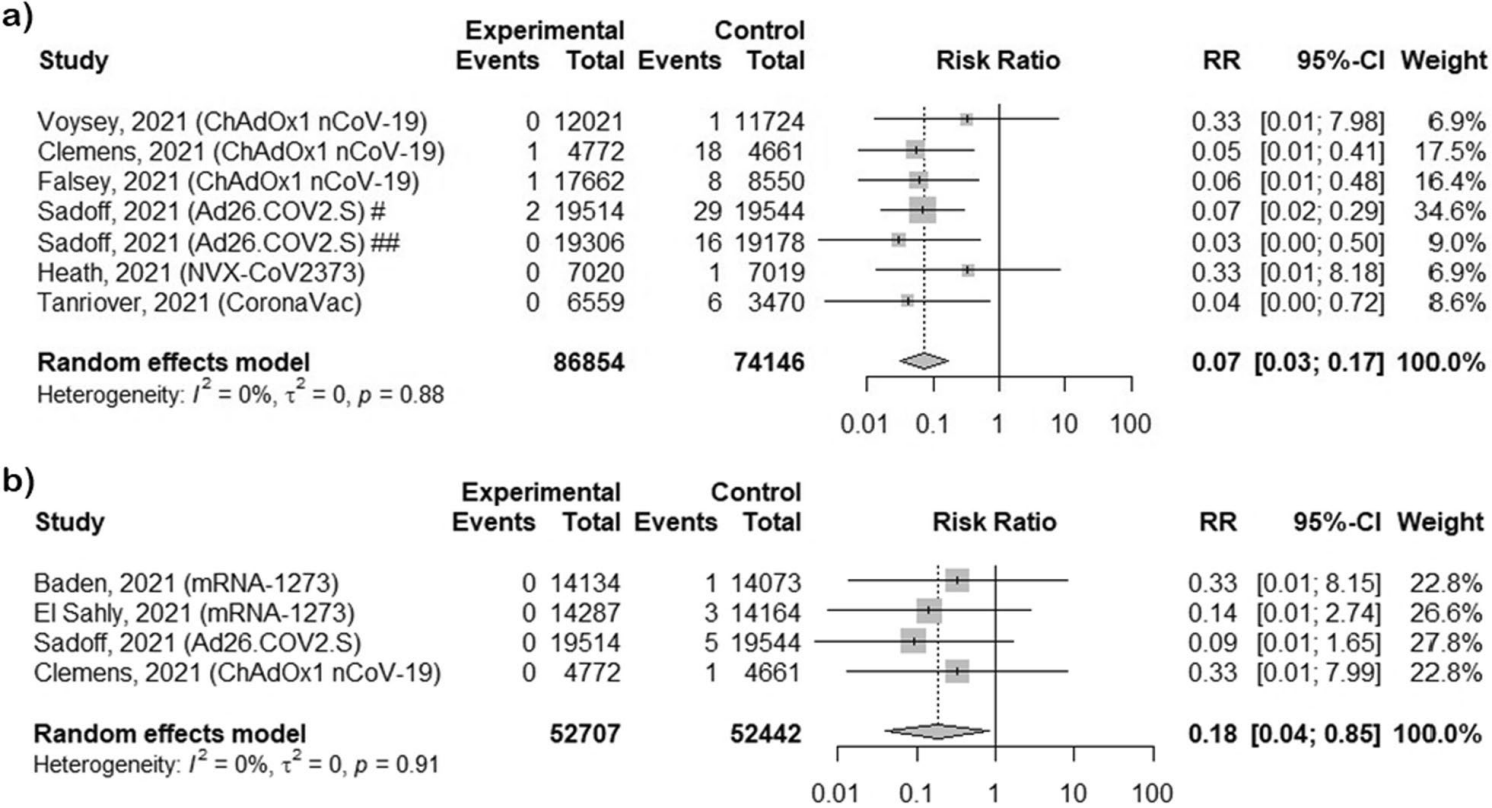
The efficacy of COVID-19 vaccines for preventing hospitalization and death related to COVID-19. **a** preventing hospitalization related to COVID-19; **b** preventing death related to COVID-19; ^#^observation at least 14 days after vaccination; ^##^observation at least 28 days after vaccination

We analyzed the efficacy of different types of vaccines in preventing hospitalization related with COVID-19 infections (except mRNA vaccines—no data has been reported), which was 93% (RR = 0.07, 95% CI [0.03; 0.17], p < 0.0001) without heterogeneity (Fig. 4a). The highest risk of hospitalization was observed after viral vector vaccine ChAdOx1 nCoV-19 and subunit vaccine NVX-CoV2373, and equals 0.33 (95% CI [0.01; 7.98] and 0.33 (95% CI [0.01; 8.18]), respectively.

### The efficacy of vaccines against death related with COVID-19 infections

Meta-analysis assessing the impact of COVID-19 vaccines on COVID-19 mortality rate was carried out in 4 clinical trials: mRNA-1273 [15, 16], ChAdOx1 nCoV-19 [23] and Ad26.COV2.S [21] vaccines (Fig. 4b). Other studies haven’t reported deaths related to COVID-19 during the study. Full vaccination may prevent death by 82% (RR = 0.18, 95% CI [0.03; 0.15], p = 0.0298) without heterogeneity.

### The efficacy of vaccines against different variants of SARS-CoV-2

**Fig. 5:**
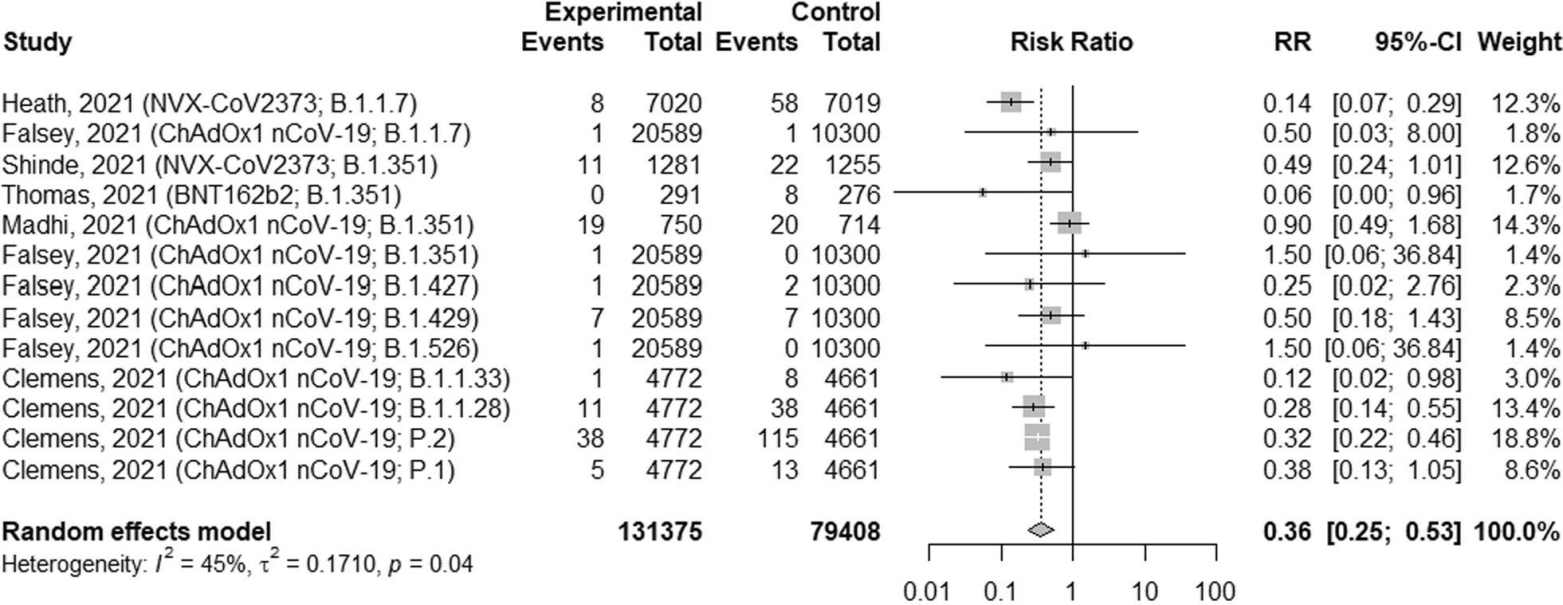
The efficacy of COVID-19 vaccines against different variants of SARS-CoV-2

Additionally, we analyzed the efficacy of several vaccines against B.1.1.7, B.1.351 variants of SARS-CoV-2 as well as against Brazilian lineages of SARS-CoV-2 (Fig. 5). Overall, full vaccination may decrease the risk of infections by 64% (RR = 0.36; 95% CI [0.25; 0.53], p < 0.0001, I^2^ = 45%). Among two analyzed vaccines (NVX-CoV2373 and ChAdOx1 nCoV-19) against B.1.1.7 variant of SARS-CoV-2, the risk of infections was the lowest after NVX-CoV2373 vaccine administration: 0.14 (95% CI [0.07; 0.29]. The efficacy of three vaccines (BNT162b2, NVX-CoV2373 and ChAdOx1 nCoV-19) were analyzed against B.1.351 variant of SARS-CoV-2. BNT162b2 vaccine may prevent the infection rate by 94% (RR = 0.06; 95% CI [0.00; 0.96]). Moreover, the risk of infections with Brazilian lineages of SARS-CoV-2 after ChAdOx1 nCoV-19 vaccine was 0.12 (95% CI [0.02; 0.98]) for B.1.1.33 variant; 0.28 (95% CI [0.14; 0.55]) for B.1.1.28 variant; 0.32 (95% CI [0.22; 0.46]) for P.2 variant and 0.38 (95% CI [0.13; 1.05]) for P.1 variant.

### The efficacy of COVID-19 vaccines against COVID-19 infections according to age groups

**Fig. 6:**
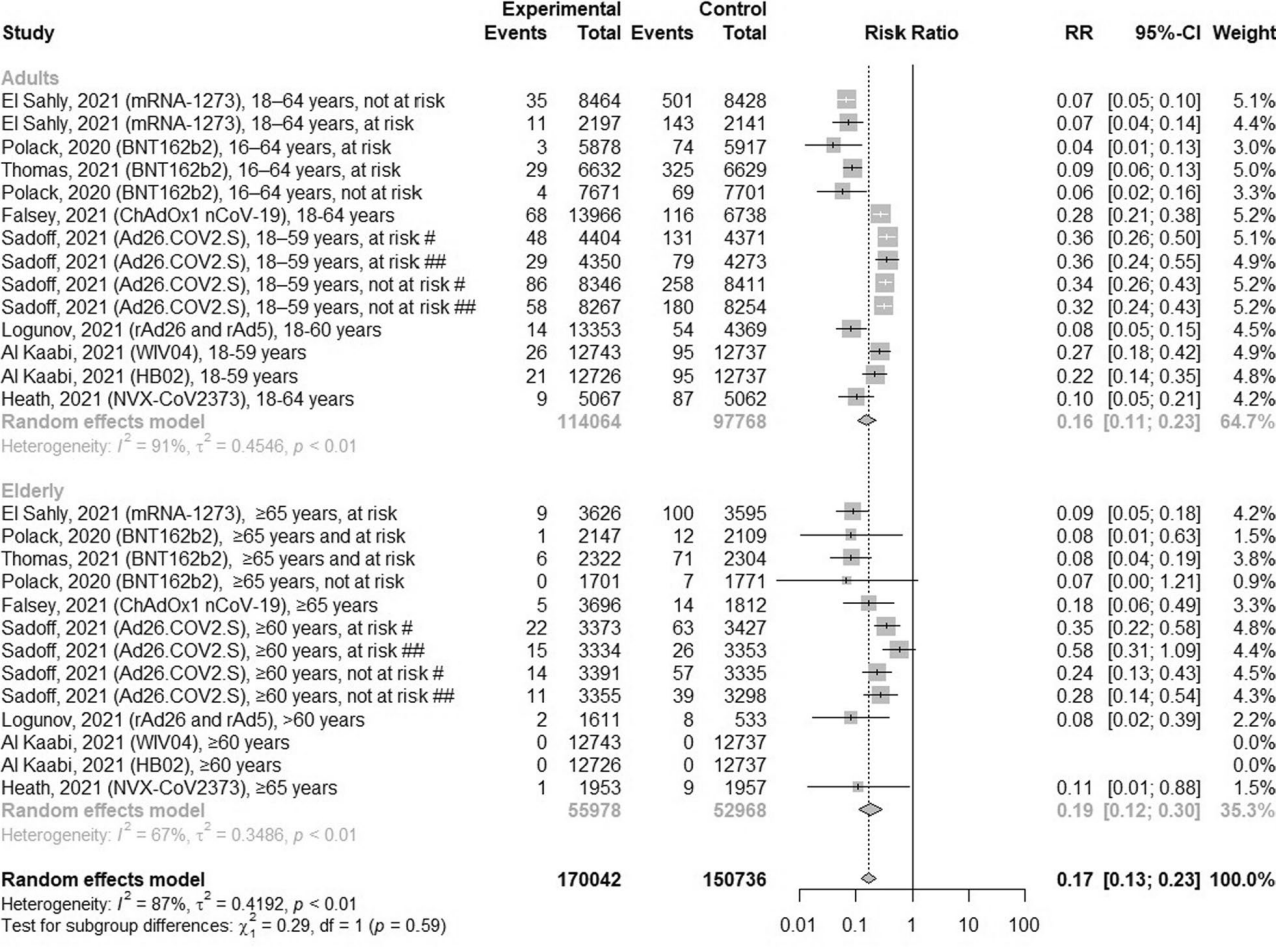
The efficacy of COVID-19 vaccines for preventing COVID-19 infection according to age groups. ^#^Observation at least 14 days after vaccination; ^##^observation at least 28 days after vaccination

According to 8 articles, we analyzed the efficacy of COVID-19 vaccines within age groups, which equaled 83% (RR = 0.17; 95% CI [0.13; 0.23], p < 0.0001, I^2^ = 87%) (Fig. 6). Moreover, the risk of the infection was slightly lower in adults compared to elderly: 0.16 (95% CI [0.11; 0.23]) and 0.19 (95% CI [0.12; 0.30]), respectively. When comparing both mRNA vaccines (BNT162b2 and mRNA-1273), the efficacy of preventing COVID-19 infection equaled 90% within all age groups. Similar result was observed after vaccination with viral vector vaccine rAd26 and rAd5 and subunit vaccine NVX-CoV2373 in both age groups. Interestingly, ChAdOx1 nCoV-19 vaccine had the better efficacy in elderly cohort $(\ge$ 65 years), because the risk was 0.18 (95% CI [0.06; 0.49]) compared to 0.28 (95% CI [0.21; 0.38]) in cohort 18–64 years.

### Publication bias

Additional file 2 shows the funnel plots for all outcomes: symptomatic COVID-19, severe COVID-19, hospitalization related with COVID-19, death related to COVID-19, different lineages of SARS-CoV-2, and according to age groups. Additionally, Peters’ regression test was performed to calculate publication bias for these outcomes. The results of Peters’ regression test showed that there was no evidence of publication bias for the association of COVID-19 vaccination and symptomatic (p = 0.1686), severe COVID-19 (p = 0.6302), hospitalizations related with COVID-19 (p = 0.9579), deaths related to COVID-19 (p = 0.2800), and against different lineages of SARS-CoV-2 (p = 0.7430), according to age groups (p = 0.5421), because *p* for outcomes was greater than 0.05.

## Discussion

Our meta-analysis sums up data from 365,744 participants from 17 randomized clinical studies of different types of COVID-19 vaccines. It shows that full vaccination could decrease the risk of symptomatic or severe COVID-19 infections, as well as the risk of death and hospitalization caused by COVID-19.

mRNA vaccines (mRNA-1273 and BNT162b2) have greater level of prevention of symptomatic COVID-19, that equals 92%. We analyzed three published articles from clinical trials of mRNA-1273 vaccine: two from phase 3 performed in the U.S. between July and October 2020 on adults with average age 51.4 years [15, 16] and one from phase 2–3 performed in the U.S. between December 2020 and February 2021 on adolescents with average age 14.3 years [17]. The vaccine successfully may prevent symptomatic infection as well as development of severeCOVID-19 infection symptoms. Moreover, the lowest risk of symptomatic infection was observed in adolescents cohort that was 0.05 and no severe cases and death have been documented in this cohort [17]. This vaccine also may prevent death related to COVID-19 in adult cohort. Unfortunately, as of the date the searching the data for analysis, there was no published clinical trial data about the efficacy of a given vaccine against different types of SARS-CoV-2. However, case–control study that was conducted in Qatar showed that the effectiveness of mRNA-1273 vaccine against B.1.1.7 variant of COVID-19 after at least 14 days after the second dose was 96.4%; whereas against B.1.351 variant of COVID-19 as well as severe or fatal COVID-19 infection was 95.7%. Additionally, effectiveness against symptomatic infection was 98.6% after at least 14 days following the second dose [31]. Moreover, in other observational study the effectiveness of this vaccine based on Cox model reached 100%, because none of cases was characterized by positive PCR test results after 14 days following the second dose [32]. The other mRNA vaccine, BNT162b2, decreased the risk of severe COVID-19 only by 75%. Moreover, no deaths related with COVID-19 were reported during this clinical study [14]. This vaccine was also effective against B.1.351 variant of SARS-CoV-2 and decrease the infection rate by 94%. The effectiveness of BNT162b2 vaccine in age group over 80 years after 14 days following full vaccination was 89%, as showed test negative case–control study by Bernal JL et al. [33]. The large study performed in Israel on around 1.2 mln participants (596,618 vaccinated and 596,618 unvaccinated participants) showed that the vaccine efficiency against symptomatic infections reached 94%, whereas against severe COVID-19 was 92% after at least 7 days following full vaccination [34]. Similar results were shown in meta-analysis of 19 observational studies: BNT162b2 vaccine reached 95% effectiveness against COVID-19 infection [35]. Moreover, both mRNA vaccines have the similar efficacy higher than 90% in adults and elderly cohorts with and without comorbidities.

In comparison to mRNA vaccines, viral vector vaccines were less effective against symptomatic COVID-19, as their efficacy equaled merely69%. rAd26 and rAd5 vaccine showed the best effectiveness to prevent symptomatic infection at level of 92%. Moreover, this vaccine can prevent severe COVID-19 infection in 99% of patients. During clinical study of this vaccine, 4 deaths occurred, but 2 of them were not associated with COVID-19 infection. However, two remaining COVID-19-associated deaths occurred 4–5 days after the first dose, despite a negative PCR test at randomization. The authors concluded that participants were already infected prior to enrollment in the study, taking into account the incubation period of infection [20]. Therefore, these data were not considered in the meta-analysis. Additionally, there were no differences in efficacy in groups distinguished by age. Other viral vector vaccine, Ad26.COV2.S, is a single-dose vaccine. We compared the efficacy of this vaccine after 14 days and 28 days after administration. Interestingly, the efficacy to prevent symptomatic COVID-19 after 14 days was slightly greater than after 28 days and equaled 67% and 60%, respectively. Conversely, in the case of severe COVID-19 prevention efficacy was estimated on the level of 77% after 14 days following dose and 85% after 28 days following dose. Additionally, this vaccine can decrease the risk of COVID-related death as well as hospitalization. The last vector vaccine, ChAdOx1 nCoV-19 had the varying efficacy score of 22–90% to prevent symptomatic COVID-19. The lowest efficacy was observed in patients infected with the B.1.351 (beta) variant, whereas the highest efficiency was observed in one of the cohorts from trial conducted in the United Kingdom, in which the first dose was applied at low concentration, while the second dose at standard concentration. Moreover, the analysis of efficacy against different variants of SARS-CoV-2 showed that ChAdOx1 nCoV-19 vaccine had low overall efficacy against B.1.1.7, B.1.351, B.1.429 and B.1.526 variants, whereas the efficacy against Brazilian variants was reached at least 62%. Moreover, the lower risk of infection was observed in the elderly cohort compared to adults.

Moreover, we analyzed the efficacy of inactivated vaccines, such as WIV04, HB02 and CoronaVac, which equals 76%. Interestingly, that the risk of symptomatic infection after CoronaVac was different based on two studies: 0.15 from Turkey and 0.39 from Indonesia, which can be explained by different extent of severity of the pandemic in these countries. WIV04 and HB02 vaccines had the same efficacy to prevent severe COVID-19 that was 80%. Moreover, no deaths associated with COVID-19 during the clinical studies of these vaccines have been documented [28–30].

Finally, subunit vaccine NVX-CoV2373, which overall efficacy was 80% against symptomatic COVID-19 infections and 84% against severe COVID-19. However, these results vary in 2 studies that were performed in different countries: South Africa where the efficacy against symptomatic and severe COVID-19 was around 60–67% and the United Kingdom where the efficacy was 90–91%. These can be explained by dominating different variants of SARS-CoV-2 in countries: in the United Kingdom most of cases had B.1.1.7 variant and the efficacy of vaccine was 86%, while in South Africa most of cases were affected by B.1351 variant and the efficacy of vaccine was merely 51%.

In summary, our meta-analysis shows that COVID-19 vaccines are effective against COVID-19. Vaccination in general reduces the risk of severe disease, which in turn minimalizes therisk of hospitalization and COVID-19-related deaths. However, our meta-analysis has some limitations. Because vaccine efficacy can be affected by factors such as the study population, study region, pandemic intensity, and vaccine type, there was considerable heterogeneity in our meta-analysis. Therefore, we used subgroup analysis by vaccine type to reduce it. Because COVID-19 vaccine development is still continued and clinical trials are still ongoing, and up to date published results are sparse, therefore only 17 studies were included in our meta-analysis. In addition, the clinical trials analyzed are preliminary because they have limited follow-up time. It is important to investigate the long-term efficacy of vaccines.

Unfortunately, our meta-analysis is not the first meta-analysis to analyze the efficacy of COVID-19 vaccines [36–38]. In addition to our meta-analysis being based on more recent data published through November 2, 2021, we analyzed the efficacy of vaccines relative to the prevention of not only symptomatic COVID-19 infections, and the prevention of severe symptoms, but also against hospitalizations and COVID-19 mortality. We also included data on vaccine efficacy in adolescents. In addition, we compared vaccine efficacy across age groups and found that vaccines have similar efficacy in adults as in elderly. Because SARS-CoV-2 virus continues to mutate and develop new variants, it is important to test the efficacy of vaccines against new SARS-CoV-2 variants. Unfortunately, as of the date we did our search, there were no published clinical trials as to the efficacy of COVID-19 vaccines against B.1.617.2 variant of SARS-CoV-2.

## Conclusions

Similarly, as in case of many virus-related diseases, in case of COVID-19, successful vaccination is the only way to maintain proper control over the disease. Therefore, the need for well-investigated, efficient vaccine is justified. In turn, to assess efficiency of vaccine candidates well-designed and properly conducted RCTs are necessary. So far, all clinically tested vaccines proven to be successful in preventing severe COVID-19 infection course as well as COVID-19 related death prevention. Further examination, including the longer period of observation and more patients recruited to the ongoing studies are still required.

## Supplementary Information

**Additional file 1. **Risk of bias of included studies.

**Additional file 2. **Funnel plots for the associations of between vaccines and COVID-19 infections.

## Data Availability

All data generated or analyzed during this study are included in this published article and its additional files.

## Abbreviations

COVID-19: Coronavirus disease 2019
SARS-CoV-2: Severe acute respiratory syndrome coronavirus 2
RR: Relative risk
