# Supplementary material for: COVID-19 vaccination efficacy in numbers including SARS-CoV-2 variants and age comparison: a meta-analysis of randomized clinical trials
Source: Ann Clin Microbiol Antimicrob. 2022 Jul 3;21:32. doi: 10.1186/s12941-022-00525-3 (PMC9250750; doi:10.1186/s12941-022-00525-3)
Supplement: Supplementary file 1 — Additional file 1. Risk of bias of included studies. [file 12941_2022_525_MOESM1_ESM.pdf]

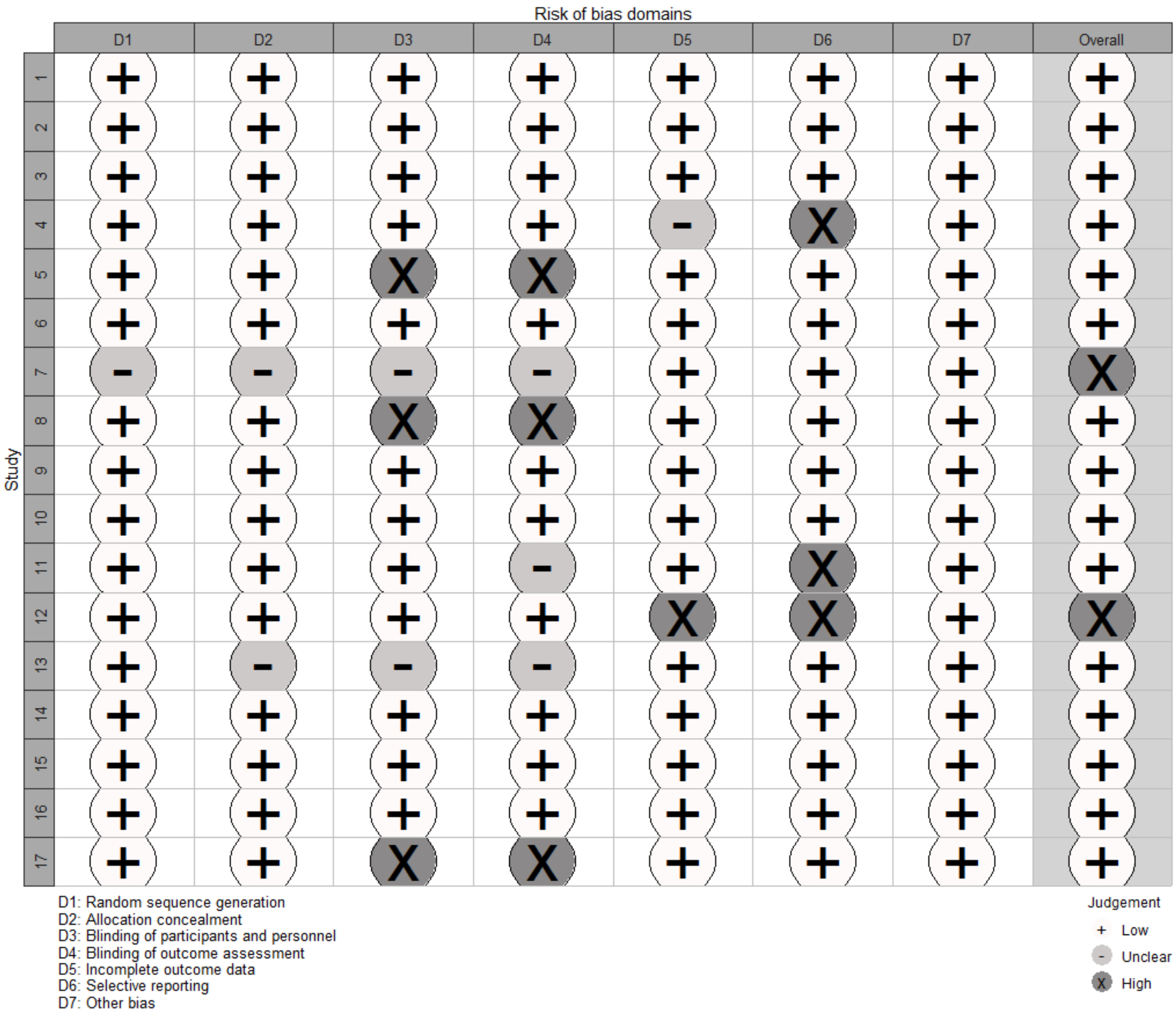

Additional file 1. Risk of bias of included studies.

(1) Baden, 2021; (2) El Sahly, 2021; (3) Ali, 2021; (4) Polack, 2020; (5) Thomas, 2021; (6) Frenck, 2021; (7) Madhi, 2021; (8) Clemens, 2021; (9) Falsey, 2021; (10) Sadoff, 2021; (11) Logunov, 2021; (12) Al Kaabi, 2021; (13) Fadlyana, 2021; (14) Tanriover, 2021; (15) Shinde, 2021; (16) Heath, 2021; (17) Voysey, 2021.
