# Supplementary figures and images for: COVID-19 vaccination efficacy in numbers including SARS-CoV-2 variants and age comparison: a meta-analysis of randomized clinical trials
Source: Ann Clin Microbiol Antimicrob. 2022 Jul 3;21:32. doi: 10.1186/s12941-022-00525-3 (PMC9250750; doi:10.1186/s12941-022-00525-3)

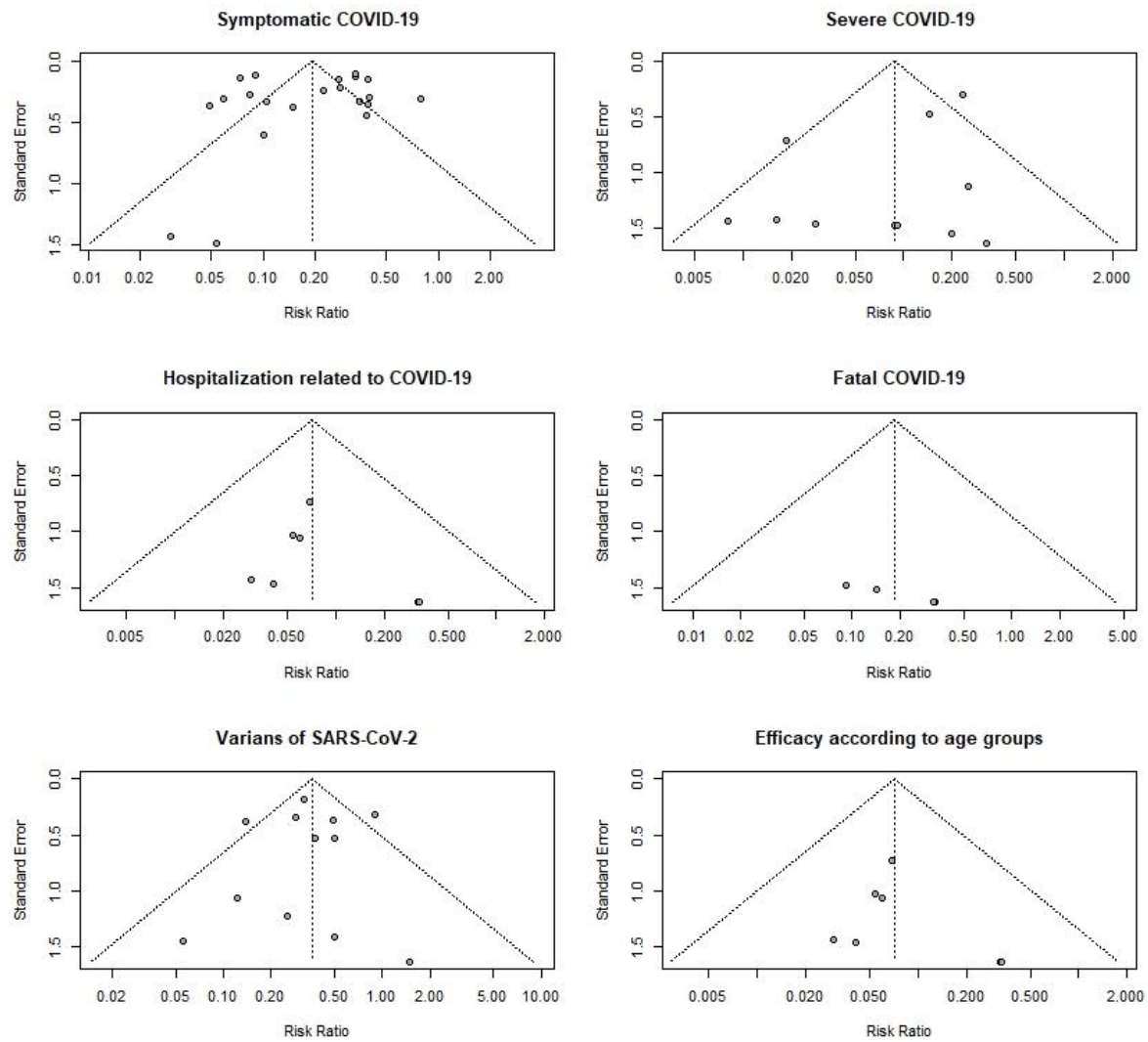

Additional file 2. Funnel plots for the associations of between vaccines and COVID-19 infections.

Supplement: Supplementary file 2 — Additional file 2. Funnel plots for the associations of between vaccines and COVID-19 infections. [file 12941_2022_525_MOESM2_ESM.pdf]
